# A Method Based on Multi-Sensor Data Fusion for Fault Detection of Planetary Gearboxes

**DOI:** 10.3390/s120202005

**Published:** 2012-02-10

**Authors:** Yaguo Lei, Jing Lin, Zhengjia He, Detong Kong

**Affiliations:** 1 State Key Laboratory for Manufacturing Systems Engineering, Xi’an Jiaotong University, No. 28 Xianning West Road, Xi’an 710049, China; E-Mails: jinglin@mail.xjtu.edu.cn (J.L.); hzj@mail.xjtu.edu.cn (Z.H.); dt_kong@163.com (D.K.); 2 State Key Laboratory of Mechanical Transmission, Chongqing University, No. 174 Shazhengjie, Chongqing 400044, China

**Keywords:** planetary gearboxes, multiple sensors, data fusion, sun gear, fault detection

## Abstract

Studies on fault detection and diagnosis of planetary gearboxes are quite limited compared with those of fixed-axis gearboxes. Different from fixed-axis gearboxes, planetary gearboxes exhibit unique behaviors, which invalidate fault diagnosis methods that work well for fixed-axis gearboxes. It is a fact that for systems as complex as planetary gearboxes, multiple sensors mounted on different locations provide complementary information on the health condition of the systems. On this basis, a fault detection method based on multi-sensor data fusion is introduced in this paper. In this method, two features developed for planetary gearboxes are used to characterize the gear health conditions, and an adaptive neuro-fuzzy inference system (ANFIS) is utilized to fuse all features from different sensors. In order to demonstrate the effectiveness of the proposed method, experiments are carried out on a planetary gearbox test rig, on which multiple accelerometers are mounted for data collection. The comparisons between the proposed method and the methods based on individual sensors show that the former achieves much higher accuracies in detecting planetary gearbox faults.

## 1. Introduction

Fault detection and diagnosis of gearboxes has been attracting considerable attention. Most investigations, however, focus on fixed-axis gearboxes in which all gears are designed to rotate around their own fixed centers [1,2]. Compared with fault diagnosis of fixed-axis gearboxes, there are not that many studies on fault detection and diagnosis of planetary gearboxes. The term “planetary gearbox” refers to a compound gear system which has a stationary or rotating ring gear, a sun gear that rotates around its own center, and several planet gears that not only rotate around their own centers, but also revolve around the center of the sun gear. Planetary gearboxes are widely used in wind turbines, helicopters and construction machinery due to their advantages of large transmission ratio, strong load-bearing capacity and high transmission efficiency [3,4]. They generally operate in a tough working environment and are therefore subject to different modes of damage [5].

Different from fixed-axis gearboxes, planetary gearboxes exhibit the following unique behaviors: (a) multiple planet gears produce similar vibrations. These vibrations with different meshing phases couple with each other; as a result, some of the excitations of multiple gear meshes can be cancelled or neutralized [6]; (b) planetary gearboxes present special spectral structures. The spectrum is typically asymmetric, that is, most of the vibration energy occurs at various sidebands of the gear meshing frequency and its harmonics [7]; (c) multiple and complex vibration transmission paths from the gear mesh points to the sensors mounted on the housing deteriorate or attenuate the vibration response of gear faults through dissipation and interference [8]. Thus, the vibration signals measured from planetary gearboxes and their spectra are more complex than those from fixed-axis gearboxes. Consequently, fault diagnosis methods working well for fixed-axis gearboxes often fail to detect and diagnose faults of planetary gearboxes.

To solve the problems of fault detection and diagnosis of planetary gearboxes, a few methods have recently been reported in the literature. Keller and Grabill [9] proposed the sideband index (SI) and the sideband level factor (SLF) to detect planetary gearbox faults and found that SI and SLF were effective only for the test cell conditions, but invalid for the on-aircraft conditions. Patrick *et al.* [10] introduced an integrated framework for on-board fault diagnosis and remaining useful life prediction of a helicopter planetary gear transmission component. Blunt and Keller [6] developed two methods based on a planet and the carrier to detect a crack in the helicopter planetary gearbox. Bartelmus and Zimroz [11] proposed a feature for monitoring planetary gearboxes under time-variable operating conditions. Barszcz and Randall [12] applied the spectral kurtosis for detecting ring gear cracks in a planetary gearbox used in a wind turbine. Samuel and Pines [13] proposed a technique using the constrained adaptive lifting algorithm for detecting gear faults in a planetary gearbox. Gao *et al.* [14] presented a method based on redundant second generation wavelet transform to diagnose a ring gear fault of a planetary gearbox used in a steel plant.

The above studies, however, only utilized the vibration information from individual sensors. It is a fact that for complex gear transmission systems like planetary gearboxes, multiple sensors provide variously sensitive or complementary characteristic information for fault detection and diagnosis. Thus, how to fuse information from multiple sensors to improve accuracy is a vital issue in fault detection and diagnosis of planetary gearboxes.

Aiming at solving the above problem, a method for fault detection of planetary gearboxes based on multi-sensor data fusion is proposed in this paper. In this method, two features are employed to characterize the health conditions of planetary gearboxes, and an adaptive neuro-fuzzy inference system (ANFIS) is used to fuse all features from different sensors. Since the two features are particularly developed for planetary gearboxes, and ANFIS combines the advantages of the adaptive capability of neural networks and the qualitative approach of fuzzy logic, the proposed method is expected to produce satisfactory results in detecting planetary gearboxes faults. The remainder of this paper is organized as follows: Section 2 introduces the method based on multi-sensor data fusion. Section 3 shows a planetary gearbox test rig and experiments conducted for testing the proposed method. Gears having four fault modes were installed in the test rig and vibration signals collected by multiple sensors under different loads and various motor speeds. Section 4 compares the proposed method with the methods based on individual sensors. The experimental results show that the proposed method is superior to the methods based on individual sensors in detecting faults of planetary gearboxes. Conclusions are given in Section 5.

## 2. The Method Based on Multi-Sensor Data Fusion

**Figure 1.**
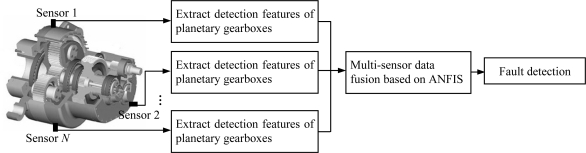
Flow chart of the proposed method.

Figure 1 displays the flow chart of the proposed method based on multi-sensor data fusion. First, features are extracted from the signals measured by multiple sensors on different locations of a planetary gearbox housing. The features are the root mean square of the filtered signal (FRMS) and the normalized summation of the positive amplitudes of the difference spectrum between the measured signal and the healthy one (NSDS). Then, all features extracted from different sensors are fused using ANFIS. Finally, the faults occurring in the planetary gearboxes can be detected according to the fusion results.

### 2.1. Two Features

As mentioned above, two features are extracted from the vibration signals of planetary gearboxes. They are FRMS and NSDS, which are specially designed for fault detection of planetary gearboxes.

#### 2.1.1. FRMS

It is generated by calculating the root mean square of the filtered signal instead of the original signal and is defined as:

(1) $$\text{FRMS}=\sqrt{\frac{1}{T}\sum_{t=1}^{T}{\left(s(t)\right)}^{2}}$$

where *s*(*t*) (*t* = 1,⋯,*T*), is the *t*^th^ data point of the filtered signal *S*, and *T* is the total number of data points contained in *S*. The filtered signal is produced by removing the regular meshing elements from the original vibration signal *X*. The regular meshing elements include the rotating frequency of the damaged gear and its five order harmonics, the meshing frequency and its three order harmonics, and the modulation sidebands of the meshing frequency and its harmonics. The modulation sidebands are excited when the planets pass through the sensors fixed on the gearbox housing [15,16].

The rationale of FRMS can be explained as follows: the vibration spectrum of a healthy planetary gearbox, measured by sensors mounted at a fixed location, is unlike that of a fixed-axis gearbox. The planet gears rotate past the sensor location causing the vibration transmission path to change in a periodic manner, thus modulating the amplitude of the measured vibration response. These sidebands are therefore the common elements in the frequency spectra of both healthy and damaged planetary gearboxes instead of the indications of faults.

#### 2.1.2. NSDS

It is developed by normalizing the summation of the positive amplitudes of the difference spectrum between the signal measured on a gearbox whose health condition is unknown and the signal measured on a healthy gearbox. It is expressed as:

(2) $$\text{NSDS}=\frac{\sum_{i=1}^{I}y_{d}\left(i\right)}{\sum_{i=1}^{I}y_{m}\left(i\right)}$$

(3) $$\left\{ \begin{array}{ll} y_{d}(i)=y_{m}(i)-y_{h}(i) & \text{if}\;y_{m}(i)>y_{n}(i)\\ y_{d}(i)=0 & \text{if}\;y_{m}(i)\leq y_{n}(i) \end{array}\right.$$

where *y*_d_(*i*) (*i* = 1, ⋯,*I*) denotes the amplitude of the *i*^th^ spectrum line of the difference spectrum *y*_d_ of *y*_m_ minus *y*_h_, *I* is the total number of spectrum lines contained in *y*_d_, and *y*_m_ and *y*_h_ represent the frequency spectra of the unknown signal and the healthy one respectively. The use of normalization, that is, dividing the sum of the positive amplitudes in the difference spectra by the sum of the unknown signal spectra, is to attenuate or even cancel the effect of the operation conditions such as various rotating speeds and loads. Therefore, NSDS is a dimensionless feature.

It is believed that the vibration response and the frequency spectra will change once faults occur in a planetary gearbox and the amplitudes in the spectra may increase correspondingly. NSDS aims to discover the increment in the frequency spectra of the faulty planetary gearbox compared to the healthy one.

### 2.2. Review of ANFIS

**Figure 2.**
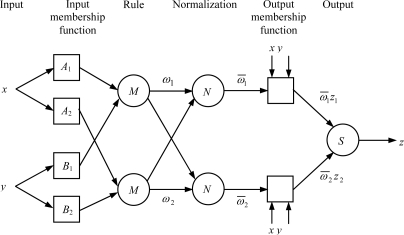
Architecture of the ANFIS.

ANFIS is an integration system which uses neural networks to optimize the fuzzy inference system. ANFIS maps inputs through input membership functions and associated parameters, and then through output membership functions to outputs. The initial membership functions and rules for the fuzzy inference system can be designed by employing human expertise about the target system to be modeled. Then ANFIS can refine the fuzzy if-then rules and membership functions to describe the input/output behavior of a complex system. To explain the ANFIS architecture, two fuzzy if-then rules based on a first order Sugeno model are considered [17–19]:

$$\begin{array}{c} \text{Rule}\;1:\;\text{If}\;(x\;\text{is}\;A_{1})\;\text{and}\;(y\;\text{is}\;B_{1})\;\text{then}\;(z_{1}=p_{1}x+q_{1}y+r_{1}),\\ \text{Rule}\;2:\;\text{If}\;(x\;\text{is}\;A_{2})\;\text{and}\;(y\;\text{is}\;B_{2})\;\text{then}\;(z_{2}=p_{2}x+q_{2}y+r_{2}), \end{array}$$

where *x* and *y* are the inputs, *A_i_* and *B_i_* are the fuzzy sets, *z_i_*(*i* = 1,2) are the outputs within the fuzzy region specified by the fuzzy rules, and *p_i_*, *q_i_* and *r_i_* are the design parameters that are determined during the training process. The ANFIS architecture to implement these two rules has five layers and is shown in Figure 2, where a square indicates an adaptive node, whereas a circle stands for a fixed node.

**Layer 1:** Input membership function

This layer performs fuzzification of the inputs and all the nodes in this layer are adaptive. The outputs are the membership grade of the inputs:

(4) $$\left\{ \begin{array}{ll} o_{i}^{1}=u_{A_{i}}(x), & i=1,2\\ o_{i}^{1}=u_{B_{i-2}}(y), & i=3,4 \end{array}\right.$$

where *u_A_i__* (*x*) and *u*_*B*_*i*−2__(*y*) can be adopted any fuzzy membership function. Usually, the bell shaped membership function is employed and given by:

(5) $$u_{A_{i}}(x)=\frac{1}{1+{\left[{\left(\frac{x-c_{i}}{a_{i}}\right)}^{2}\right]}^{b_{i}}},\;\;i=1,2,$$

where *a_i_*, *b_i_* and *c_i_* are the parameters of the membership functions, deciding the bell shaped functions.

**Layer 2:** Rule

All the nodes in this layer are fixed nodes. They are labeled with *M*, indicating that they perform as simple multipliers. The outputs of this layer represent the fuzzy strengths *ω_i_* of each rule and can be expressed as:

(6) $$o_{i}^{2}=\omega_{i}=u_{A_{i}}(x)u_{B_{i}}(y),\;\;i=1,2.$$

**Layer 3:** Normalization

All the nodes in this layer are also fixed nodes. They are labeled with *N*, indicating that they play a normalization role to the fuzzy strengths from the previous layer. The normalization factor is calculated as the sum of all weight functions. The outputs of this layer, the so-called normalized fuzzy strengths, can be represented as:

(7) oi3=ω¯i=ωi∑i=12ωi,  i=1,2.

**Layer 4:** Output membership function

In this layer, the nodes are adaptive nodes. The outputs of this layer are shown by:

(8) $$o_{i}^{4}={\bar{\omega }}_{i}z_{i}={\bar{\omega }}_{i}(p_{i}x+q_{i}y+r_{i}),\;i=1,2,$$

where *p_i_*, *q_i_* and *r_i_* are the parameters of the output membership functions respectively.

**Layer 5:** Output

In this layer, there is only one single fixed node labeled with *S*. This node performs the summation of all incoming signals. Hence, the overall output of the model is as follows:

(9) oi5=z=∑i=12ω¯izi=∑i=12ωizi∑i=12ωi

Generally, a hybrid learning algorithm of the gradient descent approach and least-squares estimate is utilized to tune the parameters of the membership functions. During the forward pass, the node outputs advance until the output membership function layer, where the consequent parameters are identified by the least-squares estimate. Based on the error signals that propagate backward, the backward pass uses the back propagation gradient descent method to update the premise parameters [17–19].

## 3. Experimental System

**Figure 3.**
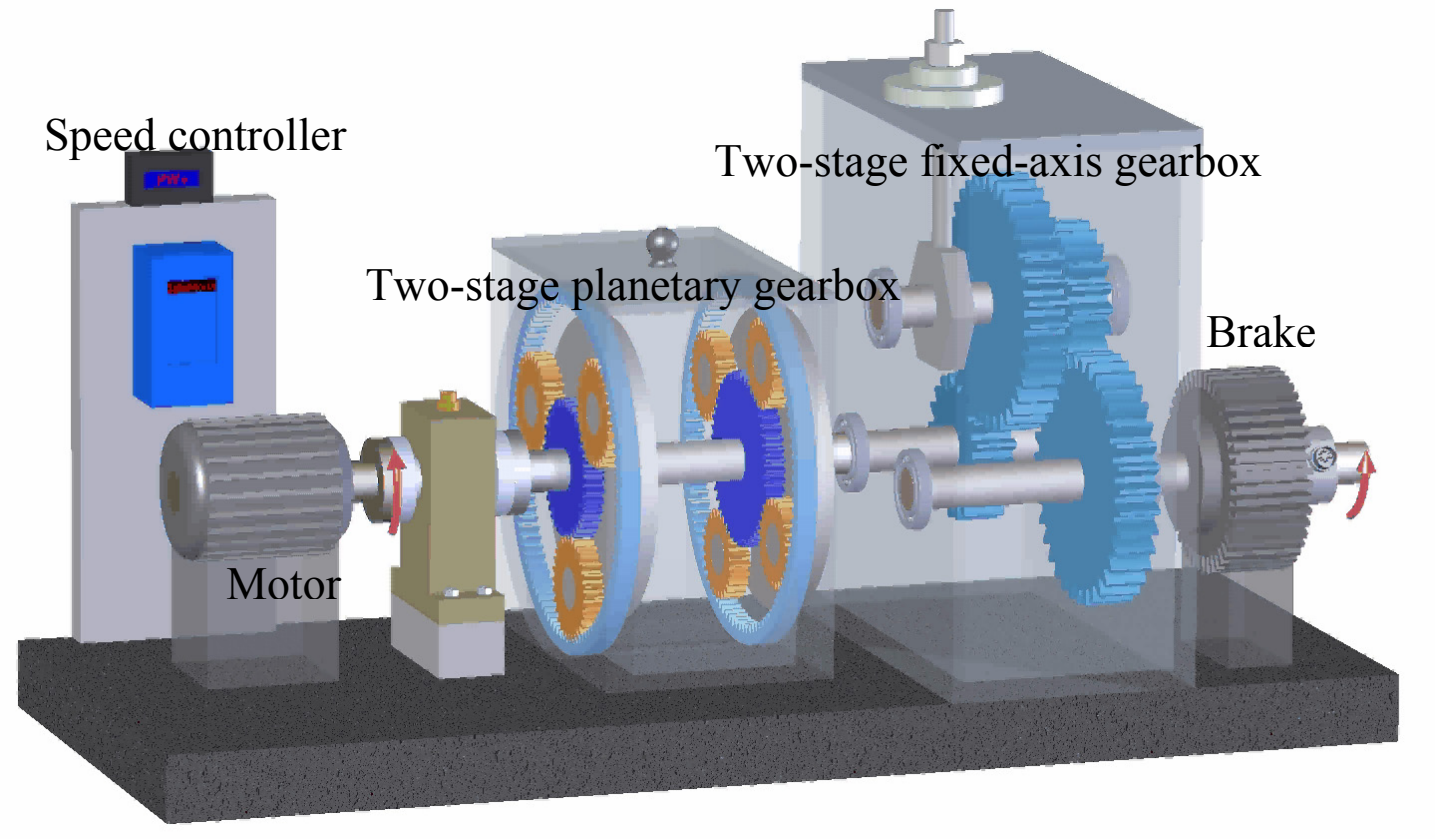
Schematic model of a two-stage planetary gearbox test rig.

In order to test the effectiveness of the proposed method, an experimental system of a planetary gearbox test rig is established and experiments are carried out on the test rig. A schematic model of the test rig is shown in Figure 3. The test rig includes two gearboxes, a 3-hp motor for driving the gearboxes, and a magnetic brake for loading. The two gearboxes contain a two-stage planetary one and a two-stage fixed-axis one. The two-stage planetary gearbox is our concern in the present study. In each stage of the planetary gearbox, an inner sun gear is surrounded by three or four rotating planet gears, and a stationary outer ring gear. Torque is transmitted through the sun gear to the planets, which ride on a planetary carrier. The planetary carrier, in turn, transmits torque to the output shaft.

**Figure 4.**
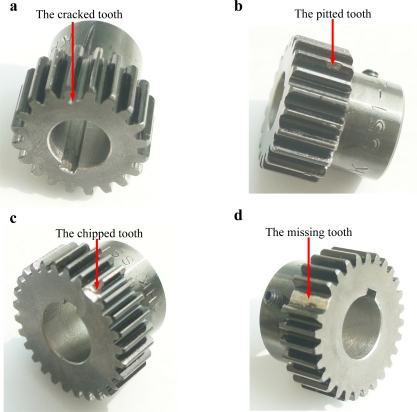
Damaged sun gears: (**a**) having a cracked tooth; (**b**) having a pitted tooth; (**c**) having a chipped tooth; and (**d**) having a missing tooth.

In a planetary gearbox, sun gear teeth are subject to faults because their multiple meshes with the planet gears increase the potential for damage [5]. Thus, a cracked tooth and a pitted tooth are created on the sun gear of the first stage, and a chipped tooth and a missing tooth are introduced inside the second stage respectively. The pictures of the damaged sun gears are given in Figure 4. Table 1 lists the gear parameters of the two-stage planetary gearbox.

**Figure 5.**
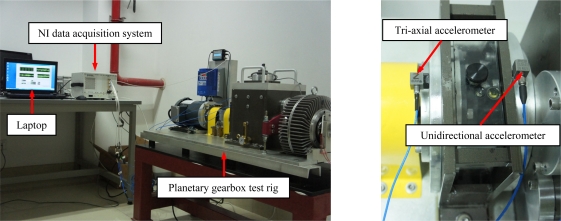
(**a**) Experimental system and (**b**) locations of the two accelerometers.

Figure 5(a) presents the experimental system, which consists of the test rig, accelerometers, an NI data acquisition system and a laptop with the data acquisition software. Two accelerometers, as shown in Figure 5(b), are used to capture the vibration signals. One is a tri-axial accelerometer mounted on the first stage bearing end cover. The other is a unidirectional accelerometer mounted on the second stage bearing end cover. All vibration signals are measured with a sampling frequency of 5,120 Hz under four different drive motor speeds (2,100 rpm, 2,400 rpm, 2,700 rpm and 3,000 rpm) and two loading conditions (no load and the maximum load).

Five experiments are conducted under each of the five gear health conditions. The five health conditions involve normal, the cracked tooth on the sun gear of the first stage, the pitted tooth on the sun gear of the first stage, the chipped tooth on the sun gear of the second stage and the missing tooth on the sun gear of the second stage. In each experiment, a sun gear of different health conditions is installed inside of the test rig, whereas all the other gears are normal. The axial and radial vibrations of the first stage are measured by the tri-axial accelerometer, and the radial vibration of the second stage is captured by the unidirectional accelerometer. The vibration signals of three channels, (*i.e.*, the axial and the vertical directions of the tri-axial accelerometer, and the vertical direction of the unidirectional accelerometer), are considered and used for multi-sensor data fusion in this paper.

## 4. Experimental Results and Comparisons

The vibration signals collected from the planetary gearbox test rig are divided into data samples. Each data sample is actually a data series containing 20,224 data points. For each identical operating condition (identical motor speed, load and gear health condition), thirty data samples are obtained. Therefore, for each of the five gear health conditions, there are 240 data samples collected under two loading conditions and four motor speeds.

### 4.1. Case 1: Fault Detection of the Sun Gear of the First Stage

In this case, the proposed method is applied to detecting three health conditions of the sun gear in the first stage of the planetary gearbox: normal, a cracked tooth and a pitted tooth. Two hundred and forty (240) data samples are acquired for each health condition, and therefore the whole data set including the three conditions altogether contains 720 samples. Three hundred and sixty samples (360) are selected for training and the remaining 360 samples are used to test. This is a three-class classification problem.

**Figure 6.**
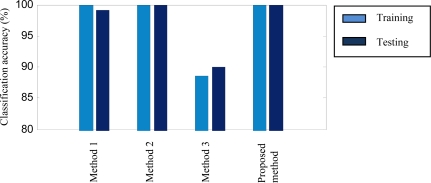
Accuracy comparison between Methods 1∼3 and the proposed method for Case 1.

First, two features FRMS and NSDS are extracted from each data sample. Correspondingly, six features are obtained for each of data samples from the three channels (the axial and the vertical directions of the tri-axial accelerometer and the vertical direction of the unidirectional accelerometer). Then, the six features are fused via ANFIS. Finally, the health conditions of the sun gear of the first stage are detected. The detection accuracies, that is, the percentage of the number of the data samples correctly detected divided by the whole number of the data samples, are presented in Figure 6 and Table 2, respectively.

To demonstrate the performance of the proposed method based on multi-sensor data fusion, the method based on each individual sensor/channel is also tested using the same data. We refer to these methods as Method 1, Method 2 and Method 3, corresponding to the three individual sensors/channels. The three methods use only two features extracted from an individual sensor/channel, and also utilize ANFIS as the fusion technique. Their detection results are also shown in Figure 6 and Table 2.

**Figure 7.**
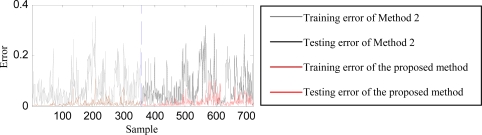
Classification errors of Method 2 and the proposed method.

Since this case is relatively simple, Methods 1∼3 achieve acceptable training and testing accuracies. They are in the range of 88.61–100% for training and 90–100% for testing. Moreover, both Method 2 and the proposed method obtain the highest training and testing accuracies (100%). Observing their classification errors plotted in Figure 7, however, we can see that the classification error of the proposed method is much smaller than that of Method 2.

### 4.2. Case 2: Fault Detection of the Sun Gear of the Second Stage

**Figure 8.**
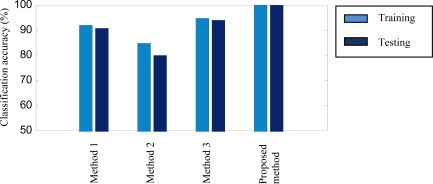
Accuracy comparison between Methods 1∼3 and the proposed method for Case 2.

The difference of Case 2 from Case 1 is that in Case 2, damage is created on the sun gear of the second stage. Similarly, 720 data samples are divided into 360 training and 360 testing samples. They are used to compare the proposed method with Methods 1∼3. The fault detection accuracies are given in Figure 8 and Table 2 respectively.

As mentioned above, faults to be detected in this case locate on the sun gear of the second stage, in which the sun gear operates under lower rotating speeds than the first stage. As a result, the fault characteristics of the damaged sun gear are not excited enough and accordingly not evident either. Therefore, it is more difficult to detect the sun gear faults of the second stage. The training and testing accuracies of Methods 1∼3 for the sun gear of the second stage in Figure 8 are obviously lower than those for the sun gear of the first stage in Figure 7. The training accuracies of this case range from 84.44% to 94.72% and the testing accuracies from 79.72% to 93.88%. However, the proposed method provides an accuracy of 100% for both training and testing. This verifies the robustness of the proposed method.

### 4.3. Case 3: Fault Detection of the Sun Gears of All Stages

**Figure 9.**
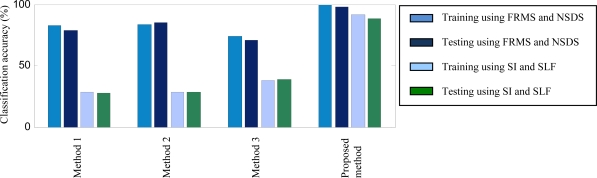
Accuracy comparison between Methods 1∼3 and the proposed method for Case 3.

The purpose of using this case is to test the performance of the proposed method in detecting both gear health conditions and damage locations (that is, damage on different sun gears of planetary gearboxes). Thus, 1,200 data samples covering five gear health conditions (normal, two damage modes in the first stage and two damage modes in the second stage) are applied to testing the proposed method. The 1,200 data samples are split into two sets: 600 samples for training and 600 for testing. In this case, we solve the five-class classification problem. Figure 9 and Table 2 display the training and testing accuracies of both the proposed method and Methods 1∼3.

It is observed from Figure 9 that the training accuracies of all methods decrease and range from 74% to 99.33% because this is a five-class classification problem and relatively difficult. But the highest training accuracy (99.33%) is still obtained by the proposed method. The testing accuracies of Methods 1∼3 are from 70.83% to 85.5% (average 78.33%), whereas the testing accuracy of the proposed method is much higher (98.33%). These imply that the method based on multi-sensor data fusion performs well when detecting not only various fault modes, but also different fault locations.

To examine the effectiveness of the two proposed features FRMS and NSDS, we make another comparison in this case. Two features SI and SLF, presented in Reference [9], are used as the inputs of ANFIS to detect the planetary gearbox faults. The detection accuracies of both Methods 1∼3 and the proposed method are also shown in Figure 9. For Methods 1∼3, the accuracies of using SI and SLF are quite low compared with those of using FRMS and NSDS. However, the multi-sensor fusion method still obtains the highest accuracy, although this accuracy is not as high as the method achieves when using FRMS and NSDS. It means that the proposed features perform better than the existing features in detecting the planetary gearbox faults.

All results of the three cases prove that it is better to fuse the data from different sensors than to utilize the data from individual ones. The multi-sensor fusion method obtains evident improvements of accuracy and robustness in fault detection of planetary gearboxes.

## 5. Conclusions

Multiple sensors mounted on different locations of planetary gearboxes can provide complementary information for fault detection and diagnosis. Based on this understanding, a method using multi-sensor data fusion is proposed in this paper. In this method, an adaptive neuro-fuzzy inference system (ANFIS) is adopted as the fusion technique and two features specifically designed for planetary gearboxes as the inputs of ANFIS. Three cases of planetary gearbox fault detection are used to test and compare the proposed method and the methods using individual sensors. The experimental results demonstrate that the proposed method is superior to the others in terms of fault detection accuracy.

## Figures and Tables

**Table 1.** Gear parameters in the two-stage planetary gearbox.

**The first stage**					**The second stage**			
Gear	sun	planet	ring	number of planets	sun	planet	ring	number of planets
Number of teeth	20	40	100	3	28	36	100	4

**Table 2.** Detection accuracies of Methods 1∼3 and the proposed method.

**Case**	**Method 1**		**Method 2**		**Method 3**		**Average of Methods 1∼3**		**Proposed method**	
	**Training**	**Testing**	**Training**	**Testing**	**Training**	**Testing**	**Training**	**Testing**	**Training**	**Testing**
1	100	99.17	100	100	88.61	90	96.20	96.39	100	100
2	91.94	90.55	84.44	79.72	94.72	93.88	90.37	88.05	100	100
3	82.5	78.67	84	85.5	74	70.83	80.17	78.33	99.33	98.33
